# Navigating the Mental Lexicon: Network Structures, Lexical Search and Lexical Retrieval

**DOI:** 10.1007/s10936-024-10059-8

**Published:** 2024-03-01

**Authors:** M. P. Agustín-Llach, J. Rubio

**Affiliations:** 1 Departamento de Filologías Modernas, Universidad de La Rioja, Logroño, Spain; 2 Departamento de Matemáticas y Computación, Universidad de La Rioja, Logroño, Spain

## Abstract

This paper examines the implications of the association patterns in our understanding of the mental lexicon. By applying the principles of graph theory to word association data, we intend to explore which measures tap better into lexical knowledge. To that end, we had different groups of English as Foreign language learners complete a lexical fluency task. Based on these empirical data, a study was undertaken on the corresponding lexical availability graph (LAG). It is observed that the aggregation (mentioned through human coding) of all lexical tokens on a given topic allows the emergence of some lexical-semantic patterns. The most important one is the existence of some key terms, featuring both high centrality in the sense of network theory and high availability in the LAG, which define a hub of related terms. These communities of words, each one organized around an anchor term, or most central word, are nicely apprehended by a well-known network metric called *modularity*. Interestingly enough, each module seems to describe a conceptual class, showing that the collective lexicon, at least as approximated by LA Graphs, is organised and traversed by semantic mechanisms or associations via hyponymy or hiperonymy, for instance. Another empirical observation is that these conceptual hubs can be appended, resulting in high diameters compared to same-sized random graphs; even so it seems that the *small-world* hypothesis holds in LA Graphs, as in other social and natural networks.

## Introduction

In the present study, we use network theory metrics to examine the mental lexicon of the Foreign Language, understood as a complex cognitive system. We believe the way the system, i.e. the mental lexicon, is organized has important implications for the process of lexical search and lexical retrieval (De Deyne & Storms 2008, Vitevich 2008, Kenett et al., 2011, Borodkin et al., 2016, Siew et al., 2019, Kumar et al., 2022, Wulff et al., 2022, Cosgrove et al., 2023). By using a semantic fluency task, we have learners produce lexical items related to some stimuli categories (cf. e.g. Borodkin et al., 2016). From these category-related association data, we are able to build learners’ own lexical networks and examine them employing the tools provided by graph theory.

The mental lexicon is stored in semantic memory in a structured and organized way. We believe that each lexical item represents a node in a network. The relations between the nodes are called edges and they represent semantic or formal similarity (Zemla & Austerweil 2022, Goñi et al., 2010, Collins & Loftus 1975) although some testing characteristics might give rise to random or erratic associations (Fitzpatrick 2007). The thicker the edges, i.e. the more their weight, the stronger the connection they represent, and thus the easier their access (Steyvers & Tenenbaum 2005, Zemla & Austerweil 2022, Wulff et al., 2022). Lexical retrieval is the process by which speakers access those lexical items. The fact that this information is organized and structured allows for efficient lexical retrieval and communication (Goñi et al., 2010) ; see also Goñi et al. (2011) for work on fluency data, and Citraro & Rossetti (2020) for the notion of modularity within semantic networks. Identification of this precise organization of the network might provide some interesting insights into the mechanisms that govern the mental lexicon, and thus how words are learned, stored and retrieved from the mind when needed, for instance, for communication purposes. In this paper, we try to approach this ideal inter-subjective mental lexicon by means of a tool called *Lexical Availability Graph* (LAG) (Ferreira and Echeverría (2010); see also e.g. Zemla & Austerweil (2022) for a review of several other methods to construct networks).

To end this introduction, the concepts from graph theory which are more relevant to our work are informally presented in Table 1; their mathematical definitions can be found in Newman (2018), Cherven (2015) , or Siew et al. (2019), for instance (see Sect. 5.3 below for the used definition of *clustering coefficient*).

**Table 1:** Description of relevant graph-related metrics (see, for instance, Newman 2018)

Metric	Description
Degree	Number of connections
Path length	Number of edges that connect one node with another one
Distance between two nodes	Length of shortest path connecting the two nodes
Average shortest-path length	Average of distances between all pairs of nodes
Diameter	Maximum of distances over each pair of nodes in the graph
Clustering coefficient	It quantifies how close neighbours are to being a *clique* (complete graph)
Modularity	Strength of division of the network into modules (groups, clusters, communities). [High modularity implies dense connections between nodes within each module but sparse connections between nodes in different modules.]

## Graph Theory and Language Studies

Semantic memory studies have strived to use the mathematical graph theory and its metrics to look into how human language is organized in an attempt to better understand how words are organized and retrieved. Banking on semantic fluency data, association norms or free association data, these studies (see e.g. Wulff et al., 2022, Kenett et al., 2011, Borodkin et al., 2016, Siew et al., 2019, De Deyne & Storms 2008), look into how healthy and unhealthy participants, younger and older speakers, and native and L2 learners organize, store and retrieve the words from their mental lexicon. Vitevich (2008), focusing on phonological organization of word-forms, and Steyvers & Tenenbaum (2005), De Deyne & Storms (2008) or Kenett et al. (2011) on lexico-semantic data, were able to determine that mean path length, clustering coefficient, degree distribution and assortative mixing were helpful in establishing some constraints that might affect lexical acquisition and word learning. They conclude that there is a big core of highly connected words and many lexical islands with nodes with fewer connections, many of which were not connected to the largest component. This, they believe, resembles the small-world structure of other social and behavioural networks. In psycholinguistic terms, this interpretation follows that some words establish many interrelationships and share many neighbours, i.e. words that have many connections (degree) tend to be connected with other highly connected words forming densely related clusters, whereas more scarcely connected words also have neighbours with few connections, thus take the form of more isolated islands. One consequence of this structure is that accessibility is quick, robust and accurate. The fact that there are plenty of alternative pathways to access a specific lexical item (clustering coefficient and degree) enables this rapid retrieval. Chan and Vitevich (2010) reached a similar conclusion in that they highlight the impact of a structure with high clustering coefficient. They looked at speech errors and again could determine that words stored together establish a dense network of links, so errors tend to appear in the words with many interconnections, i.e. the words in the cluster. They conclude that words with high clustering coefficient are more difficult to retrieve. However, it is important to highlight that connections are more often than not led by semantic (rather than phonetic) similarity (see e.g. Fitzpatrick 2007, Hernández et al., 2006). Their conclusions might be hard to place in the study of lexical-semantic networks, such as we intend to do here.

In a more general study about language networks, Solé et al. (2010) could ascertain that those language networks display two main characteristics: (a) They have a small-world structure, i.e. it is a very well connected graph, where it is easy to reach one element through a small number of jumps (high clustering coefficient and low average path length); and (b) Heterogeneity, meaning that most elements have very few connections, and some few nodes have many connections (measured by degree distribution). These are called *hubs* and are key components of web complexity and represent categories. Steyvers & Tenenbaum (2005) had already pointed out that semantic networks generated through word association patterns were characterized by sparse connectivity, low average path length and strong local clustering, with sparsely connected lexical islands hovering the dense hubs (small-world structure and scale-free associative patterns); see also Kenett et al., 2011. They argued that this structural organization reflects how the lexicon grows, namely, new words connect to the existing network via different connectivity patterns, e.g. semantic relations of different types: synonymy, superordination, metonymic relations, etc. In this sense, they state that earlier acquired words are expected to show higher connectivity and newly acquired words would show fewer connections (p. 44). Social networks and natural language networks obtained from native data have been probed to show the small-world structure mentioned above (see e.g. De Deyne & Storms 2008, Kenett et al., 2011, Borodkin et al., 2016, Zemla & Austerweil 2022). Accordingly, preliminary work on lexical availability graphs have also suggested that native language and second language data obtained in experimental conditions via the lexical availability category generation task also display the said small-world structure (e.g. Ferreira & Echevarría 2010, Salceldo et al., 2013).

In a pioneering work in the exploration of the network metaphor and graph theory, Wilks & Meara (2002) specifically used graph density to compare L1 (mother tongue) and L2 (second language) vocabularies. They found that the graphs built from native associative data display higher density figures than those conformed with learner data. Their interpretation is that native networks are more interconnected, i.e. have more nodes and more edges between them. More recent work, however, has shown that graph density is inversely related to proficiency level with lower proficient learners displaying denser networks than natives or high proficient learners (Quintanilla 2019). The different data gathering instruments might account for the contradictory results, since Wilks & Meara (2002) used a task where participants had to circle words they thought were linked between them, i.e. perception, whereas the latter studies used fluency tasks of the lexical availability type, i.e. production. The description that Wilks & Meara (2002) make of the lexical network concurs with subsequent interpretations, a highly connected small core sparsely connected or isolated nodes in less central positions. Few other researchers within cognitive science and semantic memory have contributed with studies on L2 acquisition. Kenett and his associates (e.g. Kenett et al., 2011, Borodkin et al., 2016) pursue the interest of examining mental lexicons of L2 learners via lexical networks techniques. Mainly, they try to establish the structure of the lexicon and compare L1 and L2 access, revealing that the structural characteristics of the L2 network are different from L1 networks resulting in learners having less organized and therefore less accessible lexicons.

As a conclusion on this quick tour of the using of network metaphors in language studies, we could assume, as a working hypothesis, that the mental lexicon is organised as a network where nodes are the terms and edges are associations between terms, being the nature of these associations largely undetermined, especially in L2 lexical retrieval. Our purpose in this paper is to approach this ideal network by means of a graph obtained through a fluency task. In addition, our techniques provide an approximation to a collective or inter-subjective lexical network for English as Foreign Language (EFL), as it is explained in the next section.

## Vocabulary Production and Lexical Availability in EFL as a Fluency Task

From the different models that try to represent how we store and access the mental lexicon, the spreading activation model by Collins and Loftus in 1975 (1975) seems to most closely link to the network metaphor. The lexical-semantic network is made up of nodes, representing lexical items or concepts, connected by links in pair-wise associations. Accordingly, activation spreads from node to node. This generates a graph that formally and conceptually resembles the complex networks studied by graph theory. Borge-Holthoefer and Arenas (2010) found that network topology is especially accurate in associative networks of the type proposed by Collins and Loftus (1975). Furthermore, they revealed that their structure allows for efficient retrieval and stability of the network (high clustering coefficient, short average path length, and hub-plus-islands organization), thus maximizing communication. Fluency tasks, i.e. the ease with which respondents retrieve information, are the instruments to gather data to look into semantic memory and semantic networks (Kenett et al., 2011, Borodkin et al., 2016). If semantic memory works with some structured principles, then, we believe that general structural properties of the semantic network might help elucidate those principles and how they operate in lexical learning and retrieval (cf. Steyvers & Tenenbaum 2005).

Indeed, the LAG is to be conceived as a big graph from which subgraphs or subnetworks are to be analysed. Experimental data obtained from L2 learners gives responses in chains, which are then submitted to analyses in an aggregated manner in order to build the LAG. Accordingly, the LAGs obtained are mere approximations to the big graph, which are dependent on the way data were collected, e.g. in an academic context. In addition, assumptions are made concerning the fact that they are networks shared by a social, cultural, or academic community. These LAGs are not claimed to be the mental lexicon as such, but the experimental approximations obtained via the response chains of the LA task.

A LAG is constructed as follows. In the context of their own English as a Foreign Language (EFL) classroom, L2 learners are presented with a prompt or stimulus category (for instance: *animals*) and for two minutes each learner writes a sequence of words related (in their mind) to the target prompt. From each response chain a linear graph is built, where the nodes are the word types and there is a directed edge for each pair of contiguous words in the sequence. Then, the nodes and edges of each learner are aggregated in a LAG by simply grouping all together. See also Lerner et al. (2009), Shrestha et al. (2015), Lenio et al. (2016) for a similar methodology of graph construction by simple association. Sinha et al. (2023) believe that if scale-free networks with small-world properties are produced with this method, then it is a valid method for graph construction.

Let us describe more precisely by means of a hypothetical example how the LAG is constructed. Imagine that when faced with the stimulus *animals*, an informant writes these four words, in this order: DOG, CAT, LION, TIGER. Then, with this input, a directed path is constructed, composed by the three directed edges: DOG–CAT, CAT–LION, LION–TIGER. Now, we consider it as a set of directed edges: {DOG–CAT, CAT–LION, LION–TIGER}. By aggregating all the sets of edges obtained from all the informants over the same stimulus, we get the LAG for that stimulus.

The structural properties of our LA Graphs that made them different from other network-based approaches in the literature are the following:

1. The number of nodes is not fixed or constrained in any way: the nodes are exactly the words the informants produced for that stimulus, as a set (without repetition).
2. Similarly, the number of edges is not fixed or constrained: the edges are all the pairs appearing following the procedure explained above (repetitions are allowed, this time).
3. As a consequence of points 1 and 2, the density of the graph is directly related to the input data, without any elaboration, and can be, thus, used to explore the lexical properties of the inputs.
4. The graph is directed, and then the directed edges reflect the order in which the words were written (for instance, LION–TIGER is a different edge than TIGER–LION), allowing the analyst to explore also features related to the way the words are retrieved from the mind.
5. The graph is weighted: each edge is annotated with the number of informants that generated it. This characteristic allows us to prune the graph according to a certain weight, avoiding spurious edges without much lexical information and focusing on the most relevant parts of the graph.
6. The graph can be connected or not. This permits the analyst to study the number of connected components, their sizes, how they relate to different metrics and so on.

As a qualitative criterion, we can say that this way of constructing lexical networks is little intrusive and that arbitrary decisions are kept to a minimum: the graph is defined almost directly from the input data (this is to be compared with other approaches where, to define the network, some amount of pre-processing is needed (cf. Zemla & Austerweil 2022 and Christensen & Kenett 2021 for a very thorough account of graph construction methods, and specifically, in the latter for a guide and tools to construct graphs). We are, however, not implying that the method of construction cannot have an influence on the numerical outcomes and, then, on the consequences drawn at a linguistic level. In fact, this is always the case, therefore, it is important to supervise how the methodological decisions can skew research conclusions, as we will explain along the paper.

Once the LAG is constructed, it is possible to start studying it as a complex network. In particular, the centrality of a node is an indicator of how relevant that node/word is within the network, i.e. within the mental lexicon. However, there is lack of consensus concerning what it means to be central for a specific lexical item, e.g. having more connections (average degree) (Borge-Holthoefer & Arenas 2010), being most frequent (cf. frequency studies, e.g. Meara 2009), being most available (cf. lexical availability studies, e.g. Ferreira and Echevarría 2010, Samper 2014), being easily reached from other nodes (closeness) as in Borge-Holthoefer & Arenas (2010), being on the shortest path between pairs of other nodes (betweenness) Borge-Holthoefer & Arenas (2010), having not only many but also relevant (again central) connections (eigenvector centrality and pageRank) Borge-Holthoefer & Arenas (2010). So it is important to elucidate which of these multiple centrality notions are relevant in our context of L2 LAGs.

When looking at similar works for inspiration with respect to this problem, we found that studies applying the semantic network metaphor and using graph theory analyses with second language data are very scarce. Apart from the pioneering work by Wilks & Meara (2002) explained above, we are only aware of the studies of Ferreira & Echeverría (2010), Borodkin et al. (2016) with EFL learners and Salcedo and colleagues (e.g. Quintanilla & Salcedo 2019) with Spanish FL learners. These studies focused on studying semantic associations and yielding them into graphs. Specifically, Ferreira and Echevarría in 2010 (Ferreira & Echeverría, 2010) found that whereas native speakers were able to organize their lexical items effectively into subcategories, EFL learners could only distinguish among large semantic categories and not beyond. Other studies with EFL learners (e.g Quintanilla & Salcedo 2019) constrain to the description of learners’ EFL vocabularies and their cohesion degrees and communicability. So far, we are not aware of studies with EFL data that focus on identifying graph metrics and mathematical regularities in the learners’ mental lexicon. This is the gap we intend to fill in here. Previous research on verbal fluency data have focused on native production (but see Borodkin et al., 2016), and mainly on the taxonomic category of animals (see e.g. Kenett et al., 2011, Wulff et al., 2022). The present research is novel because it addresses the semantic network of Spanish EFL learners, which has not been researched to date within network science, to our knowledge, but which makes a substantial number of potential stakeholders. Additionally, the “traditional” taxonomic category *animals* is compared and contrasted in terms of network generation with the more experiential category *countryside*, which can be believed to result in slightly different graphs since the associations established are also different. This also opens the field to new avenues of analysis and interpretation within semantic fluency and network science. It was against this background that the present research study was designed. It was conceived as an attempt to look into the mental lexicon and vocabulary production where the use of mathematical models (graph theory) allows the description of semantic organization and the extraction of regularities and principles concerning lexical retrieval.

## Objectives

1. Describe the lexical-semantic network of a group of EFL learners (that it to say, describe their group characteristics) using graph theory metrics and check whether it follows a small-world structure similar to that reported in other social networks.
2. Find the best centrality measures and the most central nodes, which might be the communicatively most efficient.

Additionally, and as a by-product of the main objectives above, we intend to examine the role of the features of the used LA task on graph results (for instance, the influence of the length of chain responses or the way in which the directed edges are built).

## Method

The present study has an experimental design with association data, so that it features high ecological validity. The key points of the design are presented below.

### Informants

Our study has been carried out with several groups of Spanish students of English as L2 in order to analyse their lexical fluency in English FL. Specifically, a group of 98 Spanish EFL learners participated in the study. They were in the final year of their Baccalaureate, the year preceding university entrance (grade 12). They were aged 17–18. All students were learners of English as a foreign language and were at the B1 level of proficiency in English (CEFR). From all the prompts presented to this specific group of students, we have selected in this paper only two (*animals* and *countryside*) for reasons made explicit in the next subsection.

### Instruments

As said before, a lexical availability (LA) task, which is a multi-response fluency task type, was used to elicit production of vocabulary data from informants. The LA presents informants with a stimulus or cue word and asks them to generate responses related to the stimulus category. In particular, learners had to write, in two minutes, as many words came to their mind (e.g. Hernández Muñoz 2014, Jiménez Catalán 2014) in relation to the prompts: *Animals* and *Countryside*. These two prompts were selected on three grounds: they feature (a) Different productivity, (b) Different response diversity or response spread and (c) Different cohesion index. *Animals* is an inclusive or closed category which gives rise to many but very homogeneous responses. *Countryside*, is a less productive prompt, but where a broader amount of types are to be found, it is more open and gives rise to more heterogeneous responses (e.g. Hernández Muñoz 2014, Tomé Cornejo 2015). Participants were instructed in Spanish L1 and each prompt and the corresponding responses occupied an independent sheet of paper. The lexical availability task collects multiple responses from learners (cf. Schmitt 1998, Jiménez Catalán 2014) and gives thus a more complete picture of learners’ lexicons (Sheng et al., 2006, Precosky 2011). Multiple-response association tests tend to prompt chain responses that associate one another rather than to the stimulus word (cf. Precosky 2011, De Deyne & Storms 2008). That is, the word produced will facilitate or prime recall of other related concepts or word forms, this is called a *priming effect*.

### Procedures and Analyses

Informants completed the lexical availability task in class as a pen and paper task. Responses were then typed into computer-readable form for each of the prompts. The data were carefully edited, adopting the following criteria:

1. No repetitions per informant were allowed,
2. Spelling errors were corrected,
3. Multiple word response were hyphenated in order for them to be counted as a single word (e.g., fresh-air).

Once the editing process was complete, the data were typed into text files. Data were processed by means of the Gephi software package (Cherven Cherven 2015). See also Zemla & Austerweil (2022), Borodkin et al. (2016), Christensen & Kenett (2021), for alternative ways to construct graphs. This program Gephi allows to construct graphs from association data and obtain different key statistical measures, such as, for instance, average degree, clustering coefficient, diameter, eigenvector centrality, or closeness to mention but a few. Due to the importance of the concept in our work, the notion of *clustering coefficient* computed by Gephi needs to be explained in more explicit terms. Given a vertex *v* of a graph, we call *clustering coefficient of the vertex* *v* the density of the full subgraph expanded by the adjacent vertices to *v*. Numerically, if *k* is the number of adjacent vertices to *v* and *r* is the number of edges in the expanded subgraph, the clustering coefficient of *v* is $r/k(k-1)$ (recall that our graphs are directed, and then $k(k-1)$ is the number of edges in the directed clique with *k* vertices). Then, the *clustering coefficient* of the graph is the average ranging over the vertex set of the clustering coefficient of each vertice.

Additionally, data were submitted to Dispogen (Echeverría et al., 2006), a tool which allows to obtain the lexical availability index of individual words. This LA index is calculated by taking into consideration both the frequency of appearance of the items and the position where they appear, i.e. whether they are recalled first, second, third and so on. LA index can well be considered a centrality measure. The exact formula it uses to calculate the availability of a specific word is the following (see Callealta & Gallego 2016).

$$\begin{aligned} D(P_j) = \sum _{i=1}^n e^{-2.3(\frac{i-1}{n-1})}\frac{f_{ji}}{I_1} \end{aligned}$$

where:

- $n =$ maximal position reached by the word in the sample,
- $i =$ position of the word at the specific test explored,
- $j =$ target word index,
- $e =$ Euler’s number (Napier constant),
- $f_{ji} =$ absolute frequency of word *j* in position *i*,
- $I_1 =$ number of informants in the sample,
- $D(P_j) =$ target word’s *j* availability.

Data are analysed in an aggregated fashion as it is common with this type of studies. Although individual variation is important in word associations, there is enough overlap in the responses so that many responses are shared by the informants (see e.g. Wulff et al., 2022 for individual networks and their comparison to aggregated ones). This allows us to make general claims about the LAG and the approximation to the collective mental lexicon it purports. This technique has been chosen since it best fits the goals of the present study (cf. Zemla & Austerweil 2022, p. 54).

## Results

The first objective of the present study was to identify the structure of the Foreign Language (FL) mental lexicon through the examination of the approximations given by the LAG obtained experimentally; specifically, to check whether it followed a small-world structure comparable to other social and natural networks. For that purpose, we constructed graphs with our data and looked at their metrics. It was difficult to interpret results about clustering coefficients and average path lengths to determine whether the small-world structure holds for two main reasons:

1. Lack of other experimental data and studies deriving from FL learners which would allow us to compare our figures with others, and
2. The limited size of our own data.

To solve this problem, we decided to generate random graphs, by means of Gephi, with the same number of nodes and roughly the same density (i.e. around the same number of edges) as our original experimental graphs and then use them as the base for comparison.

In Table 2 we summarize some important figures about the *Animals* and *Countryside* graphs (and the corresponding random graphs).

**Table 2:** Some metrics about *Animals* and *Countryside* graphs

	Animals	Countryside
Nodes	240	431
Edges	942	918
Density	0.016	0.005
Mean clustering coefficient	0.19 0.02 (random)	0.034 0.004 (random)
Average shortest-path length	3.879 3.173 (random)	5.689 2.786 (random)
Diameter	13 9 (random)	20 8 (random)

From the data shown in Table 2 some conclusions can be drawn. First, as expected, *Countryside* defines a more disperse graph than *Animals*, with same number of edges but almost twice as many nodes, providing, therefore, a much smaller density (indeed, these figures explain that the lengths of the chain responses are equivalent on both topics, because the number of edges is almost the same in both cases). Clustering coefficient is much smaller in *Countryside* than in *Animals*, too. And finally, the diameter explodes in *Countryside*, illustrating the looser interactions among terms in this topic.

When compared to the corresponding random graphs, it is clear that the clustering coefficient is higher in the experimental graphs (one order of magnitude in both cases: multiply by 10 the random coefficient). Diameter is larger than in random graphs (slightly in *Animals*, and more marked in *Countryside*). Average path lengths are also larger than in random graphs, but not in a significant way. This is characteristic for small-world graphs. We have checked that these differences hold in others datasets (with the same student group but different prompts, and also with other groups of students) (see also e.g. Kenett et al., 2011, De Deyne & Storms 2008).

**Fig. 1:**
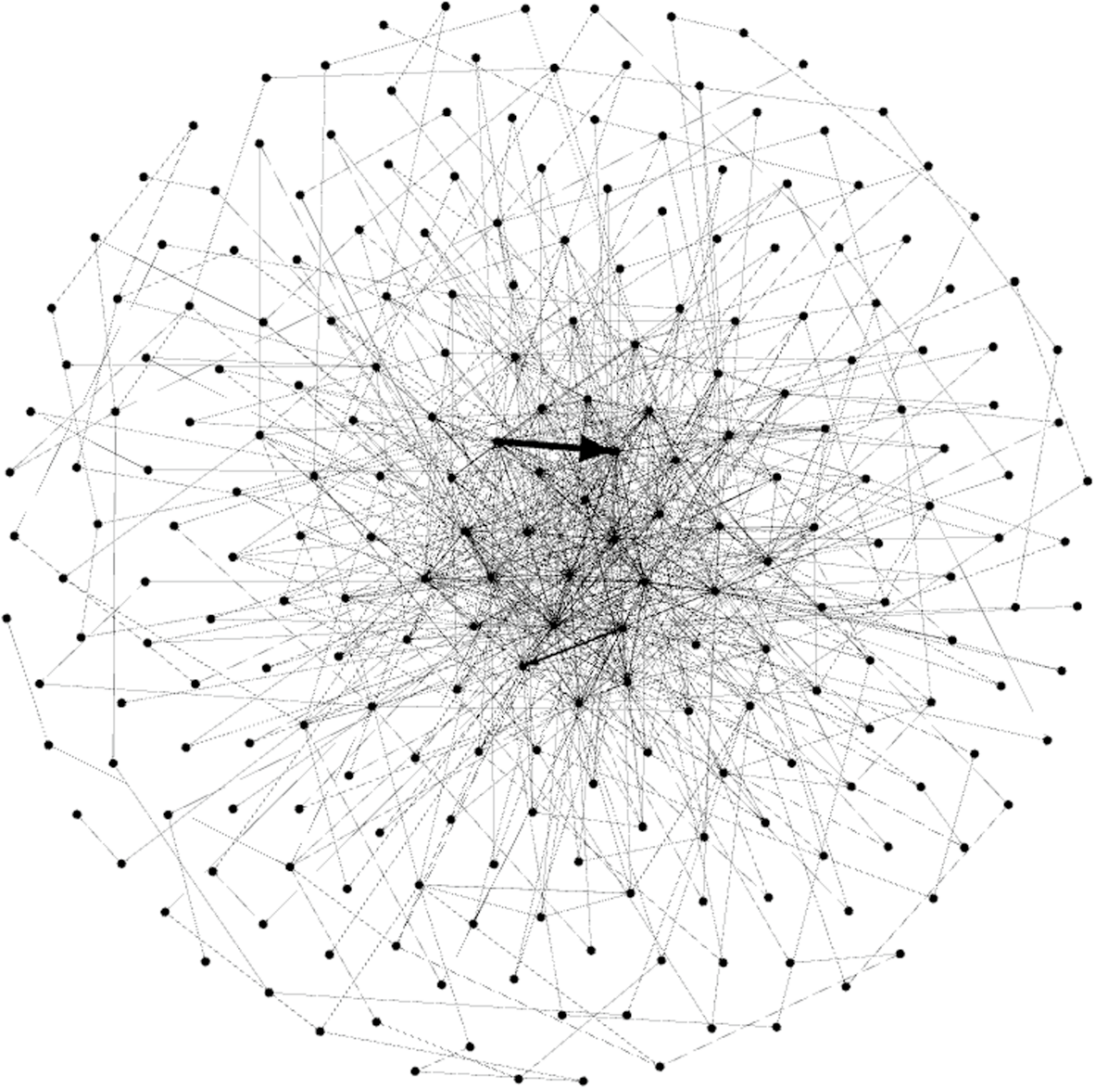
Gephi capture. The *Animals* graph is displayed by means of the Fruchterman–Reingold distribution (number of nodes: 240, edges: 942, modularity: 0.316)

**Fig. 2:**
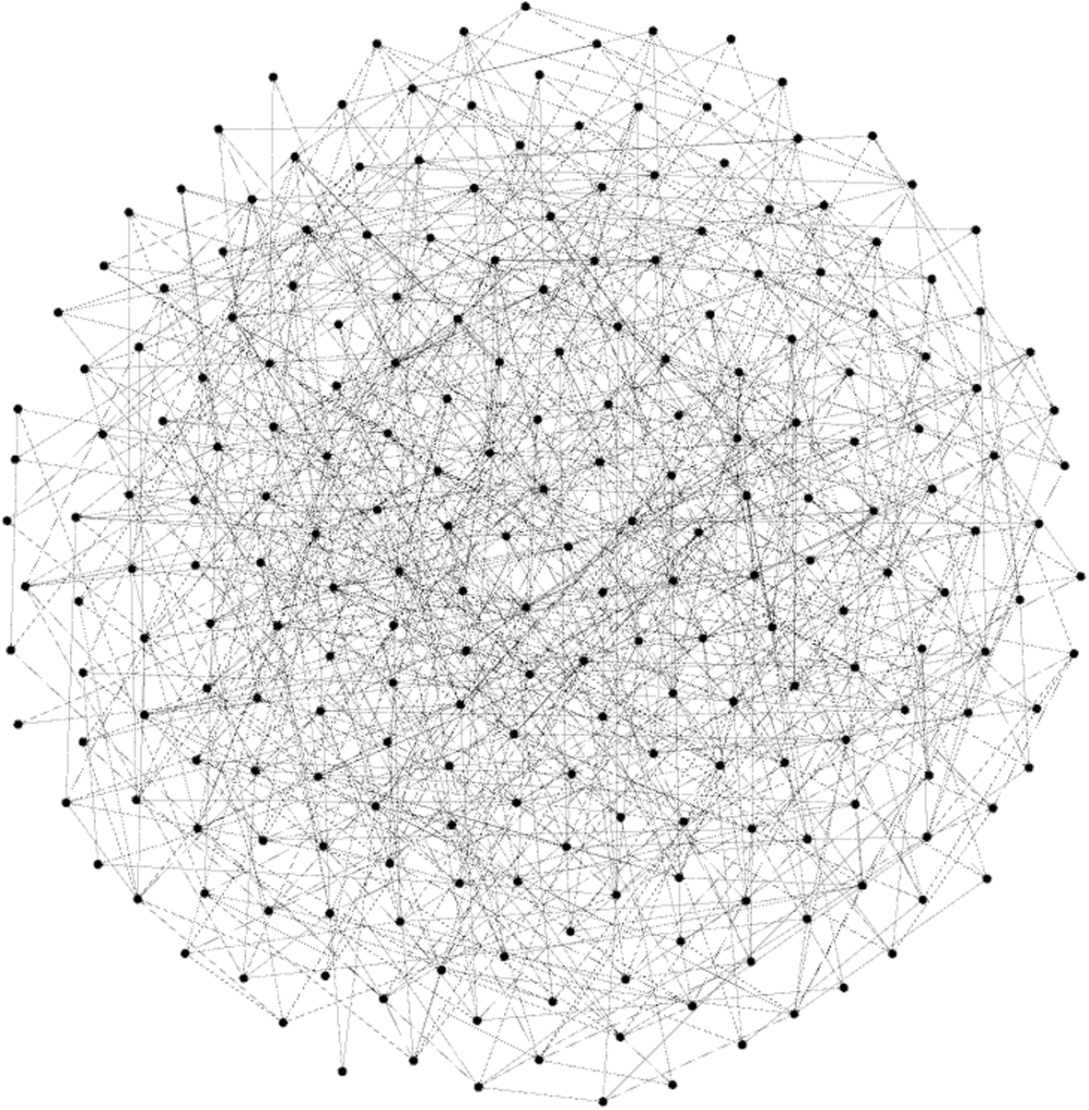
Gephi capture. A random graph with a similar number of nodes and density as *Animals* (number of nodes: 236, edges: 939, modularity: 0.326)

**Fig. 3:**
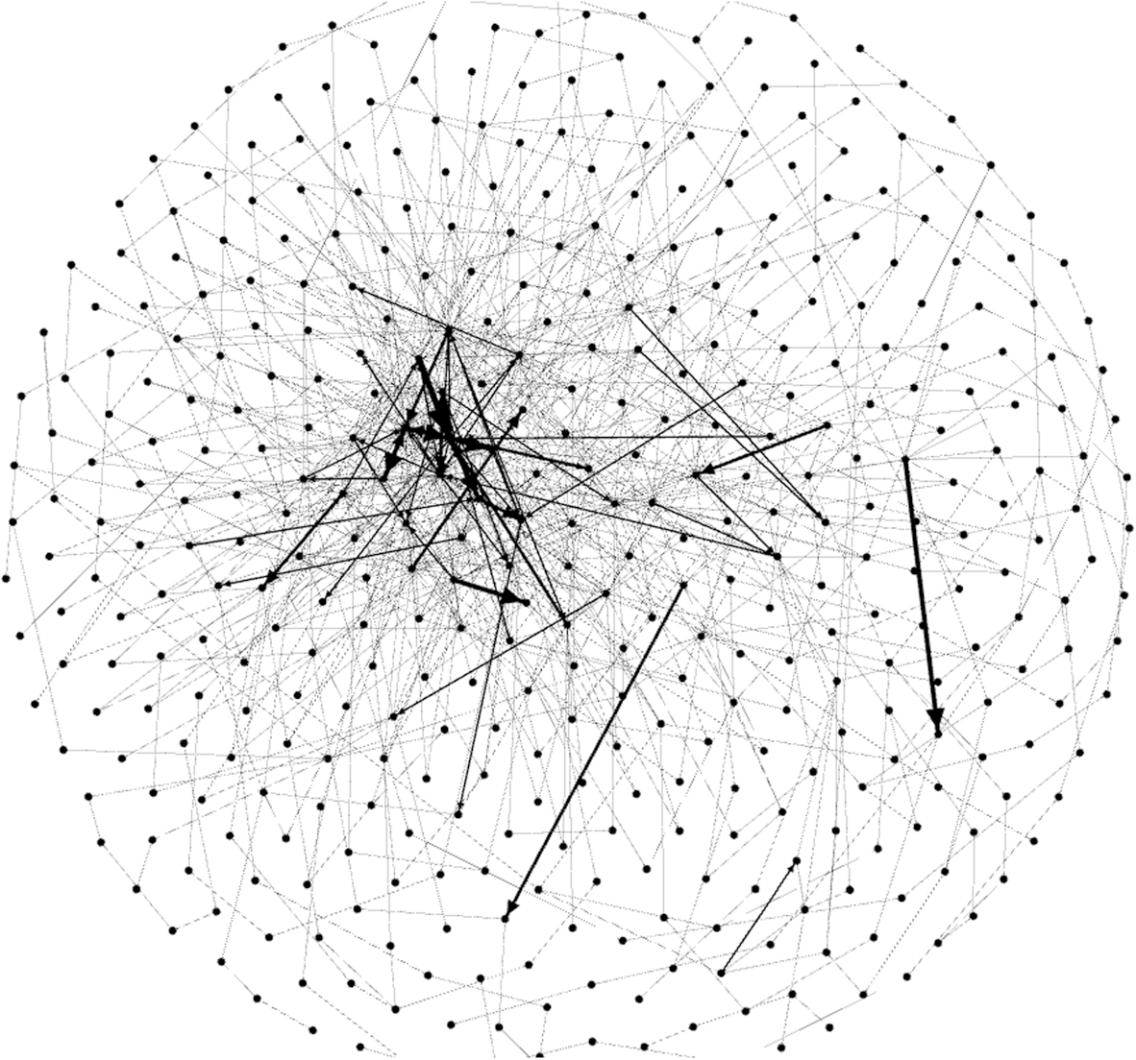
Gephi capture. *Countryside* graph (number of nodes: 439, edges: 918, modularity: 0.516)

These conclusions can be graphically observed in Figs. 1, 2 and 3, where some Gephi captures are displayed. It is visually striking how the three graphs are different, showing the dissimilar nature of them.

Our conclusions confirm the prevalence of small-world structures in linguistic networks, but diameters seem to be longer than usual. We wonder whether the way LA graphs are constructed could be producing this effect. Recall that a directed edge is provided for each pair of contiguous words in a learner’s chain response. But some of these edges could appear by some erratic reason, without expressing any lexical-semantic or formal association. Perhaps these no-sense edges are producing lengthy paths in the graph, increasing the diameter. This result is consistent with previous findings that some associations are nor based on similarity but are erratic or random (see e.g. Fitzpatrick 2007), and are congruent with the optimal foraging theory and random walks (see e.g. Siew et al., 2019), which state that learners’ resort to semantic clusters in their responses, but when the cluster is exhausted, they switch to another cluster or semantic set to continue the response chain. This switch between two semantic sets might account for those associations that are difficult to explain. For instance, when a learner exhausts the the semantic subfield of “farm animals” and switches or changes to another subfield, such as “sea animals”, we might find a bigram, an association like: “pig-shark”, which might be difficult to account for and could be termed as *random*.

**Fig. 4:**
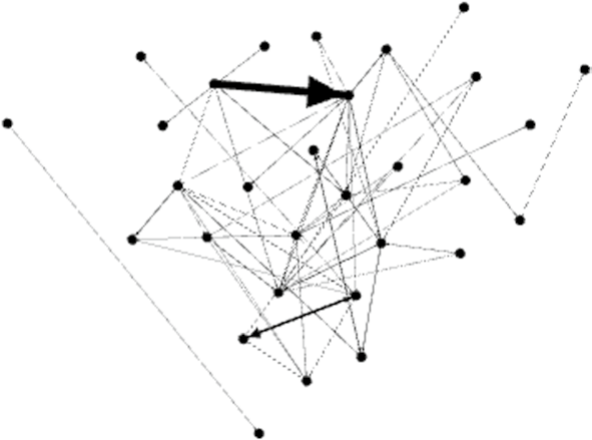
Gephi capture. *Animals* graph after pruning the edges with weights 1 and 2 and removing isolated nodes (number of nodes: 30, edges: 67, modularity: 0.415)

**Fig. 5:**
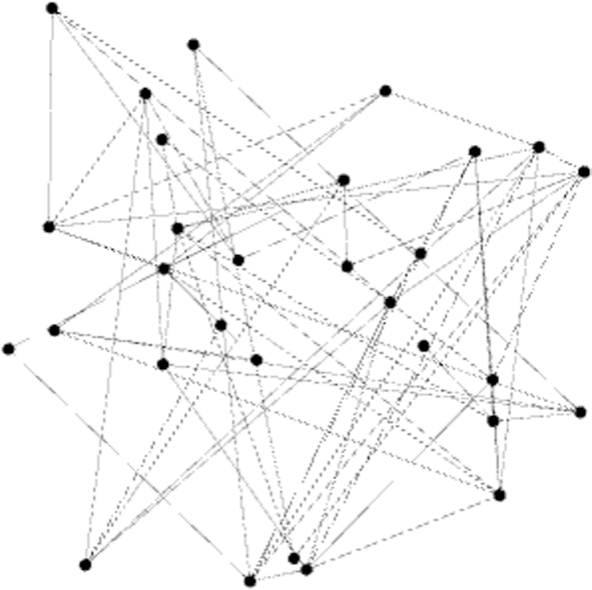
Gephi capture. A random graph with the same number of nodes as the pruned graph for *Animals*, and with a similar density (number of nodes: 30, edges: 66, modularity: 0.315)

In a trail to check the exact nature of path length, we pruned the *Animals* graph removing each edge with weight lesser than or equal to 2, and then removing the isolated nodes produced by this pruning process. In that way, we are considering only the pairs of words that have been written at least by three students, avoiding the occurrence of edges produced by chance due to the definition of a LAG. The outcomes are presented in Fig. 4 (the *Animals* graph pruned up to 2) and Fig. 5 (the corresponding random graph). We show in Table 3 the main measures from these two networks. And the conclusions stay: high clustering coefficient (0.209 versus 0.058 in the random graph) and high diameter (8 versus 4). These findings are coherent with those in De Deyne & Storms (2008) and Kenett et al. (2011).

**Table 3:** Some metrics about the pruned *Animals* graph

	Pruned animals
Nodes	30
Edges	67
Density	0.077
Mean clustering coefficient	0.209 0.058 (random)
Average shortest-path length	3.258 1.913 (random)
Diameter	8 4 (random)

Looking for a confirmation of the different nature of the networks for *Animals* and for *Countryside*, a reviewer suggested that we explore the degree *assortativity* of the networks. To this aim, we used Gephi again. This software system includes a way of computing degree assortativity. Specifically, the Gephi algorithm is based on the paper Zhang & Peixoto (2020). Following that paper, Gephi computes two values for each network:

- The *Description Length (DL)*, which measures the amount of information required to describe the network, and
- The *Number of Communities (NC)* with respect to the correlation among nodes.

We have computed these values in our running examples, and in its corresponding random counterparts, getting the following outcomes:

- *Animals*: DL 3200 approx., NC 8 (for the random network with the same number of vertices and similar density: DL 6368 approx., NC 51).
- *Countryside*: DL 5108 approx., NC 14 (random: DL 6333, NC 49).

These results are coherent with our previous findings. On one hand, both random graphs (even with different numbers of nodes and densities) have a similar behaviour between them, and, besides, with bigger DLs and much greater number of communities than linguistic graphs. On the other hand, the network for *Countryside* is more dispersed and with a greater number of communities than the corresponding one for *Animals*.

Henceforth, our results lead us to believe that the LAG of EFL learners displays a somehow regular structure, even if keeping a small-world structure. These regularities have to do with the existence of some elements, nodes, words, which are especially central or prominent in the web, and which are absent from other network types. These anchor words or *key players* (note that our use of this term does not reflect Borgatti’s(2006) approach, but it is used in the general sense of “most important members”, which here are identified with “those of higher centrality”; it is closer to the *facilitating hubs* in Kenett et al. (2011) terminology) feature high clustering coefficients, since they have a high number of neighbours clustering around them. Besides, they stand as separate entities or hubs (hubs of anchor words). Identifying these key players and the best fitting centrality measures is our second main objective. Key players in the network are relevant because if removed the network is fractured and splits. They are also key in retrieval (see Castro et al., 2017, pp. 10–11; Kenett et al., 2011).

In order to identify those key players or anchor words, we had to check for the most adequate centrality measure. Analyses revealed that when different centrality measures are calculated the word lists obtained show high degrees of overlap. In Table 4 we present the most central words in the *Animals* graph, according to several measures computed by Gephi (see Cherven 2015 for the mathematical definitions): weighted degree, eccentricity (interpreted as a measure of de-centrality, i.e. lower eccentricity means higher centrality; here, those nodes with eccentricity less than 1 are ruled out, because they are considered outliers), closeness (with values over than 1), harmonic closeness (with values over than 1) and betweenness (with values over than 1). In each box, words are sorted from highest to lowest centrality.

**Table 4:** Most central words in *Animals* (overlapping words in bold)

Weighted degree	**CAT, DOG, BIRD, FISH, LION, SNAKE, ELEPHANT, HORSE, TIGER, CROCODILE**
Eccentricity	**CAT, DOG, BIRD, LION, SNAKE, ELEPHANT, HORSE, TIGER, CROCODILE**, DOLPHIN, RABBIT, MOUSE, **FISH**
Closeness	**BIRD, CAT, SNAKE, FISH, HORSE, ELEPHANT, TIGER, CROCODILE**, MONKEY, **LION**
Harmonic closeness	**BIRD, CAT, SNAKE, FISH, HORSE, ELEPHANT, TIGER, CROCODILE**, MONKEY, **LION**
Betweenness	**BIRD, SNAKE, FISH, HORSE, CROCODILE, LION, ELEPHANT, CAT**, DOLPHIN, BEAR

Table 4 shows in an appealing way that central words in *Animals* are central...with respect to *any* centrality measure, exhibiting a great robustness regarding the importance of these terms harnessing this particular topic. But, is it a general fact or something intrinsic to the *Animals* stimulus? To try to answer this question we have repeated this calculation with a number of other prompts and groups of students. In Table 5 we cover the case of *Countryside*. As we already know, *Countryside* is a much more dispersed topic than *Animals*; then, *Countryside* is a good test case to study whether the centrality measures have a behaviour comparable to *Animals*. Bindings are looser in *Countryside*, some central words should be weaker than in *Animals*; for instance, the word with maximal degree in *Animals* is *cat* with degree 155, while in *Countryside* is *animal* with degree 66.

**Table 5:** Most central words in *Countryside* (overlapping words in bold)

Weighted degree	**ANIMAL**, TREE, RIVER, **MOUNTAIN**, GRASS, **BIRD, FLOWER, GREEN**, HOUSE, **NATURE**
Eccentricity	**COUNTRY, ANIMAL, FARM, VILLAGE, PEOPLE**, SPAIN, FIELD, MOON, CITY, TOURIST
Closeness	**GREEN, VILLAGE, FARM, PEOPLE, MOUNTAIN, WATER, COUNTRY**, HILL, **FLOWER**, FARMER
Harmonic closeness	**GREEN, MOUNTAIN, VILLAGE, FARM, BIRD, FLOWER, NATURE, WATER**, FOREST, **PEOPLE**
Betweenness	HOSPITAL, FREEDOM, DOG, BORING, TOWN, ITALY, PICNIC, ENVIRONMENT, USA, FRIEND

Table 5 makes explicit some patterns, which are frailer than those of *Animals* as it was to be expected, indicating that centrality metrics are quite compatible (except from *betweenness centrality* which seems to measure some different kind of features).

Additionally, we used the software system called Dispogen (Echeverría et al., 2006) to compute the *availability index* in both graphs. Table 6 collects the most available terms, sorted from highest to lowest index. Again, one can check that the same words appear replicated there.

**Table 6:** Most available words

	Most available words
Animals	Cat, Dog, Horse, Bird, Lion, Fish, Monkey, Snake, Giraffe,Tiger
Countryside	Tree, Animal, Spain, River, Flower, People, Town, Cow, City, Bird

These results lead us to conclude that we could choose any centrality measure to compute the anchor terms in our LAGs. Facts being like this, we select *degree centrality* as a privileged metric, because its interpretation (and definition!) is the simplest one.

Once the most central items were identified, the next step in our inquiry is disentangling the way they relate and their role in the global and local organizations of the graph. In order to avoid noise in our setting, we start exploring the pruned *Animals* graph presented above (and displayed in Fig. 4). Our first intuition was that by gradually removing less weighted edges and looking for the connected components of the corresponding graphs, some natural clusters would emerge around the anchor terms (the words with higher degree) (cf. Kenett et al., 2011 for a different but comparable procedure to calculate central hubs based on centrality and path length calculations). This would determine the “hub” connected to each harness term. Nevertheless, our calculations show that connected components are not the good metric: a big connected component appeared together with some very small residual components. By examining more closely the different structures computed by Gephi, we observed that *modularity* is the right notion. The modules (also called communities or clusters) defined by means of the modularity algorithm provide some enlightening outcomes (unsurprisingly, these modules are coherent with the topology of the network: words in a concrete module belong to the same connected component).

Table 7 gathers the clusters defined by modularity in the *Animals* graphs pruned up to 2. The reading of the table is as follows: the 5 modules in the graph are presented in each row; the first column contains, in capital letters, the anchor of that cluster (that is to say, the member of the community with highest degree); in each second column the rest of the words in that cluster are sorted from highest to lowest degree (i.e. less central terms appear at the end of the sequence).

**Table 7:** Modularity clusters in the *Animals* graph pruned up to 2

CAT	Dog, Bird, Horse, Cow, Pig, Sheep, Rabbit, Mouse, Eagle, Goat
LION	Elephant, Tiger, Snake, Crocodile, Giraffe, Monkey, Snail, Zebra
FISH	Shark, Dolphin, Turtle, Bear, Hamster
INSECT	Fly
DOMESTIC	Wild

In an unexpected and striking manner, we see how some concepts or conceptual scenarios emerge in each row. Respectively, we can identify domestic animals, wild animals and, it appears, aquatic animals, together with two small clusters, which are well-defined conceptually, but of residual size. These were the two aforementioned very small connected components.

Two remarks are worth being made. First, more powerful or central anchors or facilitating hubs (Kenett et al., 2011) define bigger hubs, i.e. 11 terms in the first row, 9 in the second one, 6 in the third and 2 in the last two rows. This can be represented by a physical metaphor: heaviest anchors attract more terms in their “gravitational basin”. Second, the links inside each conceptual scenario become weaker when words are less central; for instance, the occurrence of *eagle* in the neighborhood of *cat*, of *snail* around *lion* or *bear* and *hamster* in an aquatic cluster. Continuing with our gravitational metaphor, these less heavy elements are loosely attracted to a central point.

Again, we ask ourselves if this nice conceptual classification, emerging from the rough data, since there was no predetermined design to get this kind of clustering, could appear by chance or, perhaps, from the very taxonomic nature of the topic *Animals*, where, in addition, we have removed noise by pruning infrequent associations. Thus, we repeat our calculations with the whole topic *Countryside* without pruning it. Observe that this introduces two different kinds of difficulties. One comes from the more diffuse nature of *Countryside* (with respect to *Animals*) and the second one comes from the fact that any edge is considered, even those appearing only once, which could have been generated in a haphazard way. In Table 8, the results for *Countryside* are presented. Here, we have only considered the 30 most central words (recall from Table 2 that the *Countryside* LAG had 431 nodes), because 30 was the number of nodes in the pruned *Animals* graph. The less articulated nature of this *Countryside* graph is illustrated because 13 clusters appear (with respect to the 5 clusters in the pruned *Animals* graph), although only 7 occur among the 30 most central nodes and then Table 8 consists only of 7 rows.

**Table 8:** Modularity clusters in the whole *Countryside* graph

ANIMAL	Bird, Nature, Farm, Dog, Horse, Cow, Pig, Fresh-air, Silence, Cottage, Farmer
TREE	Mountain, Grass, Flower, Green, Forest
RIVER	Fish, Water, Lake
HOUSE	
VILLAGE	People, Country
RELAX	Peace, Family
SUN	

It is clear from Table 8 that the same patterns emerge as in *animals*, but, accordingly, in a less sharp manner. Some conceptual schemata can clearly be located in the clusters (animals and nature in the first row, mountain schema in the second one, water in the third and so on), although concepts become blurred when degree decreases. Besides, the main features noted for *Animals* are reproduced. Thus, heavier nodes attract, in general, more terms to their communities but with certain hesitations when attraction is weaker (12, 6, 4, 1, 3, 3 and 1). The behaviour regarding the cohesion of the clusters is also present in Table 8; for instance *fresh-air* and *silence* could be more related to the cluster anchored by *relax* than to that attracted by *animal*. Ours is not the first study to highlight the importance that hubs play in the networks representing the mental lexicon. Thus, Hills et al. (2009) found that hubs are central in child lexical acquisition in the L1 via a mechanism called *preferential acquisition* which is driven by the connectivity of the words (hubs of related words) in the learning environment. On a study on multiplex networks, Stella (2020) found that hubs of words with high network centrality lay at the core of the adult semantic network. Our study here proves that these high centrality words are also relevant to the EFL mental lexicon

These findings make us wonder why *snail* appears in a cluster together with jungle animals, for instance. This situation somehow resembles the situation one finds when interpreting the outputs of deep learning models. We see the final result of the algorithm but it is hard to excerpt the reasons producing it. Of course, the lack of explanation is caused by very different strands. In the case of deep learning, it is the complexity of the updating of weights in the neural network what occludes our understanding, while in the fluency task the difficulty lies in the lack of certainties in the literature concerning the mental retrieval mechanisms that link one lexical item to another. It is true that, in a given chain response of one English learner, we could conjecture why *snail* is near *giraffe*, perhaps due to some anecdotal fact. For instance, the knowledge of other languages (first, or third) might be influencing the associations because these two words could be, let us say, phonetically similar in the known languages; or maybe because of some familiar or experiential reason. Here, we need to stress that this unexplained fact is obtained after a process of pruning (so, after removing erratic associations) and an aggregation of several dozens of students responses. Therefore, there is something really unrevealed in this kind of emerging conceptual clustering. Thus, possibly some research on eXplainable Lexical Availability is needed, mimicking the flourishing field of eXplainable Artificial Intelligence.

## Discussion

Finding out the structure of the mental lexicon of learners through the approximation via LAGs can offer interesting insights into the navigation potential of the lexical-semantic web. The high clustering coefficient and high diameter of the LAGs clearly show the existence of some regularities in the network. These are identified as anchor words or words which are central to the network. We used degree as a measure of centrality after probing that different measures give similar results with degree being the most straightforward of them and easiest to grasp. This result relates to previous findings that demonstrated that the degree of a node is a good indicator of how important a given node is with regards to information retrieval and navigation within that system (Chan & Vitevich 2010, p. 686; Kenett et al., 2011). This suggests that those central anchor words help navigate the network, are more available and consequently can be retrieved more easily and quickly (cf. Steyvers & Tenenbaum 2005, pp. 69, 71; Kenett et al., 2011); and probably have been acquired earlier in life (cf. De Deyne & Storms 2015, Hernández Muñoz et al., 2006). Accordingly, if these words are removed from the network, from the mental lexicon, it can fracture and navigation is hampered.

This finding strengthens our belief that there are some key players or anchor words which are relevant for the navigation of the mental lexicon. These might correspond to prototypes (cf. Higginbotham 2010, Shivabasappa et al., 2017), firstly learned words (Hernández Muñoz et al., 2006; De Deyne & Storms 2008), most frequent words in the input (cf. De Deyne & Storms 2008; Meara 2007), emotional or affective value of the words (Van Rensbergen et al., 2016), or concreteness (Van Hell & de Grot 1998).

Additionally, we could consider modules like hubs which are made up of a group of related words and which again evolve around some central elements or anchor words. Other lexical items in decreasing order of centrality, i.e. of relevance, are linked to the core and together conform the module or hub. This observation is compatible with (Collins and Loftus 1975) on Quillian’s spreading activation theory (Quillian 1962, Quillian 1967), which advances in decreasing gradient in indirect proportion to the accessibility of the nodes. Responses given by informants are less and less relevant, but nevertheless related and accessed either directly or via the anchor word and its associates, in a sort of powerful context mechanism. These hubs have shown to be further apart, with higher diameter values as we saw above, than expected either in the same-sized random graph which lead us to detect some regularities in the structure of the network. Maybe the way in which data were collected, with chain-like responses, influences the high diameter outcome.

Results for the semantic category *Animals* are very clear, whereas for *Countryside*, they are more diffuse, as it was to be expected. In the more diffuse (less dense), less closed, and less taxonomic field of *Countryside*, more modules are found than in *Animals*. This points to *Countryside* as a semantic category where categorization is more difficult and more heterogeneous concurring thus with previous findings from categorization studies (cf. Tomé Cornejo 2015). This matches previous findings related to the fact that taxonomic categories display higher levels of organization than more thematic ones, which are less structured (cf. Chaffin 1997; Peña et al., 2002, Sheng et al., 2006). It seems reasonable to argue that access might be easier and quicker for *Animals*, since this “animal catalogue” is pre-existing in the learners’ mental lexicon, and probably has been acquired earlier in life (cf. Tomé Cornejo 2015, pp. 295, 324). On the contrary, *Countryside* is a more abstract, disperse semantic category which favours a higher number of response types (more heterogeneous or less coincidental responses) coming from this and other categories, e.g. also from animals, food, emotions, and different situations or events. Nevertheless, results are consistent and the conceptual fields identified concur with previous findings with clustering methodology (Nelson 1992). Nelson (1992) identified groups within the category *animals* which very accurately match our findings: domestic animals, wild animals, and sea animals.

As was stated before, there are other very interesting and complementary approaches within graph construction such as Zemla & Austerweil (2022), or Samuel et al. (2023) (also Stella 2020), who developed a multiplex network construction, which very much allows for a comprehensive view of the mental lexicon network. The fact that our data comes from chain-like associations produced by L2 learners who have acquired the language in a school context mainly by means of memorization or rote learning techniques and in a relatively isolated and little communicative way might account for big diameters and sparseness of connections between the different clusters (cf. Schur 2007). The way in which vocabulary and concepts have been acquired and the revious shared and individual linguistic and extra-linguistic experience might be playing a role in the regularities appearing in the structure of the LAGs.

Additionally, and as a way to link our study to other works dealing with semantic fluency data and network analysis (cf. Sinha et al., 2023), we were interested in probing whether the LAGs display the small-world structure found in other social and L1 associative networks. Our results clearly point that it is the case, confirming previous studies where small-world graphs could be identified for L1 (Steyvers and Tenenbaum 2005; Beckage & Colunga 2016; De Deyne & Storms 2015) and L2 data (Ferreira & Echeverría 2010; Sinha et al., 2023).

On a more speculative stance one could wonder why the structured patterns observed in our examples appear. A possible research path, based both on the outcomes of our experiments and on the nature of the LA task used to collect data, comes from a parallelism with respect to Turing’s notion of morphogenesis (Turing 1952). Until Turing’s paper it was believed that patterns in biological systems appear due to the stability of the processes. Turing’s breakthrough consisted in remarking that patterns and regularities are born in nature due to singularities and unstable phases: order arises from inhomogeneities. Could this phenomenon, at least heuristically, explain the patterns found in our lexical availability graphs ? Turing modelled his results through reaction-diffusion differential equations expressing chemical processes. In our case, the process is generated, by each subject, as a sequence of words. The first written word acts as a harness; from it, the subject navigates their mental lexicon retrieving words related to the first one. This is the “reaction phase”. When the “energy” of the first anchor is consumed, a “diffusion phase” starts, getting a new (less strong) harness and repeating the process, until time or knowledge are exhausted. It is important to stress that there is no mark indicating, in a collected sequence of words, when the transition from the “reaction” phase to the “diffusion” phase occurs. This implies that in the LAG there will be edges generated by “reaction” and other spurious ones (or, at least, more loosely significant), which are not describing a neat linguistic relationship, but the fact that two words are contiguous in a given sequence. It is necessary to glance up and analyze the aggregate data in the whole network to detect the patterns present in the collective lexical map. More research would be necessary to establish whether morphogenesis is a sensible explanation tool or it is simply a colorful metaphor.

Indeed, this metaphor links to two previous findings concerning: (a) Lexical availability strings, and (b) Network analysis of word association data. Concerning the first, Ávila Muñoz and Sánchez in (2011) analyze lexical availability word strings via fuzzy sets theory and argue in terms of prototype theory that respondents access the lexical network through the prototype and progressively move further away from the nucleus by retrieving less and less prototypical words until a re-entry move happens and another high prototypical item is produced, i.e. as if the respondent goes back to the core or center. Again the idea of centrality crops up in this theory since, this prototype is understood as a central item in the mental lexicon. The second main finding that links to our results and the morphogenesis metaphor pertains to network analysis which found that lexical networks are organized around hubs of a few highly connected words which facilitate search and retrieval (De Deyne & Storms 2015). (This interpretation is also consistent with optimal foraging and random walks proposed by Abbott et al., 2015 and taken up by other researchers, see e.g. Siew et al., 2019). Hubs are found to be weakly linked among themselves. These nodes in the hubs are considered to be central, and here centrality is defined as high in-degree. Our results concur with these previous findings in the identification of anchor words or words which contribute to harnessing the lexicon, anchor L2 lexical learning, and facilitate navigation. These are characterised by having many links and links to other anchor words as well. Further research should try to determine what are the lexical characteristics of these (highly accessible) words: high frequency, early-learned, concrete, emotionally-relevant or experientially-relevant (both on a group basis or on an individual basis).

##  Conclusions and Further Work

The study reported in this paper was motivated by a curiosity to explore some implications of the use of mathematical tools in the analysis of the mental lexicon and its underlying structure. It builds on previous research, which applied graph theory metrics to association data to compare the vocabularies of different FL learner populations. Two main ideas stand out in the present study. First, we could find some regularities in the organization of LAGs, even if it responds to small-world structures. When experimentally generated LAGs were compared to same-sized, same-density graphs generated randomly, we could attest high clustering coefficient along with high diameter measures for the former.

Second, anchor words or key elements in the network were identified thanks to degree as an outstanding centrality measure. Additionally, modules or hubs were also obtained which spin around the anchor words, which in turn act as magnets for other related (less central) words. Hubs are spread along the network with sparse connections among them. These hubs also allow to establish conceptual groupings used by learners to categorize the world around them, e.g. wild animals, domestic animals for *Animals* or mountain schema and water related schema for *Countryside*. The study has two main limitations as well. The first concerns the small samples which somehow limit the mathematical and technological possibilities. The second relates to the fact that we are using L2 data generated via association chains obtained in a restricted academic context. Learners’ L2 proficiency, learning context and data collection conditions might be having an undetermined effect.

In addition, we should explore the notions related to *assortativity* Zhang & Peixoto (2020) more in depth. Specifically, we should compare assortativity-communities with the modularity-communities found in this study. Since Gephi does not provide facilities for this kind of study, we should look for another tool or design our own programs to undertake this comparison.

As for further research we could try to apply deep learning to confirm or refute the findings of this paper. A first attempt to train a deep learning *language model* (Bender & Kolles 2020) (the authors are very thankful to Dr. Jónathan Heras for helping us with this development) did not give any relevant insight. The reason was the scarce data in each LAG, which forces us to mix together words elicited from different promtps and groups of students, in order to obtain a bigger dataset, losing in that way the specificities of our approach. A new perspective would be necessary to apply profitably deep learning techniques to inform L2 lexical studies. For instance, deep learning programs can be fed with both corpus-data and word-associations data to help predict learners/users’ responses to certain lexical items. This advancement can have enormous repercussions if applied to L2 pedagogy, since it can help elucidate which words to teach together or in which order to select lexical items for teaching purposes.

To end this paper, we need to emphasize, in sum, that graph theory offers a new and helpful mathematically rigorous tool to enhance the understanding of the cognitively complex mental lexicon system. However, caution must be exerted that LAGs are an imperfect approximation to try to replicate the structure of the mental lexicon. In this vein, we consider our contribution as a methodological one. Rather than offering closed solutions, a collection of tools is described in such a way that other researchers could undertake their own lexical investigations. This study is, therefore, exploratory in nature and no claims to exhaustiveness are made.
